# A non-toxigenic *Corynebacterium diphtheriae* biovar Belfanti isolated from a patient with rheumatoid arthritis in China: Insights from a comprehensive genome-based analysis

**DOI:** 10.1097/MD.0000000000042467

**Published:** 2025-05-16

**Authors:** Kai Chang, Huajie Xie, Ming Liu, Wanlin Na, Youde Liu, Qin Jia, Wei Sun, Yanyan Wang, Yuan Liu

**Affiliations:** a Department of Laboratory Medicine, The General Hospital of Western Theater Command, Chengdu, Sichuan, China; b Radiological Department, The General Hospital of Western Theater Command, Chengdu, Sichuan, China.

**Keywords:** Belfanti, *Corynebacterium diphtheriae*, genome, virulence gene

## Abstract

**Rationale:**

Diphtheria is caused by infection with *Corynebacterium diphtheriae* and is typically associated with upper respiratory tract involvement, which can manifest with high invasiveness. However, in recent years, non-toxigenic *C diphtheriae* strains have increasingly emerged as major pathogens responsible for invasive diseases. The uniqueness of this case lies in the challenges of culturing and identifying the bacterial strain, its atypical upper respiratory tract manifestations (which are easily overlooked clinically), and its potential for covert transmission, all of which warrant heightened clinical attention.

**Patient concerns:**

A 71-year-old female patient with rheumatoid arthritis presented to the respiratory department of cough with shortness of breath for >2 months.

**Diagnoses:**

Pulmonary infections caused by *Proteus vulgaris* and *C diphtheriae* biovar Belfanti, and pulmonary interstitial fibrosis.

**Interventions:**

The patient received intravenous infusion of cefoperazone sodium and sulbactam sodium. Concurrently, doxofylline was prescribed. The patient continued to receive aerosol inhalation of Budesonide Suspension for Inhalation and levosalbutamol hydrochloride.

**Outcomes:**

The patient had improved chest tightness and shortness of breath, no other special discomfort, and stable vital signs after physical examination.

**Lessons:**

*C diphtheriae* biovar Belfanti strain was isolated. The strain can’t decompose nitrate, no virulence gene and drug-resistance gene, but it has strong transposing ability and infection ability, and has the possibility of transforming into a pathogenic strain, which needs to be concerned.

## 1. Introduction

Diphtheriae, a once-common, potentially fatal infection of the upper respiratory, is usually caused by *Corynebacterium diphtheriae* (*C diphtheriae*). The symptoms of systemic infection caused by diphtheria toxin are more complex. Its forms include skin and invasive infections.^[1]^ The causative agent of diphtheria is *C diphtheriae*, which belongs to the phylum Actinobacteria. The tox gene is carried by lysogenized corynephages encoding diphtheria toxin located within the chromosome of some *C diphtheriae* strains.^[2]^ However, there is little research on the microevolutionary dynamics between tox-positive and tox-negative strains. *C diphtheriae* has a high genetic diversity and differences in its pathogenic properties. Although 4 major biovars are classified, including Mitis, Gravis, Intermedius and Belfanti, their phylogenetic relationship is unclear.^[3]^

Because *C diphtheriae* spreads among susceptible populations, mortality is high in unvaccinated young children. Although vaccines against *C diphtheriae* have existed for a long time and infants can be combined with other vaccines, such as Diphtheria-Tetanus-Pertussis vaccine, sporadic cases or small diphtheria outbreaks are inevitable, especially in areas with low or no vaccine coverage.^[4]^ Typical diphtheria is often caused by strains producing diphtheria toxin. Nontoxigenic *C diphtheriae* strains can be recovered from a variety of infections, including respiratory infections, skin infections, and bacteremia. Recently, the *C diphtheriae* biovar Belfanti isolate was considered to represent a novel species named *C belfantii*.^[5]^ Different *C diphtheriae* can be distinguished by means of biochemical characteristics. Whereas biovars Mitis and Gravis contain the diphtheria toxin gene, biovar Belfanti is rarely described as toxigenic, and intermediate bacteria is rarely isolated.^[6]^ The biovar Belfanti is not easy to be found, and there are few pathogenic reports. Transmission in the population is easily overlooked.

The aim of the study is to analyze the possible clinical symptoms and genomic characteristics of the biovar Belfanti through this case combined with whole genomic technology to provide a reference for subsequent laboratory diagnosis and preventive treatment research.

## 2. Case presentation

### 2.1. Medical history and symptoms

**Figure 1.**
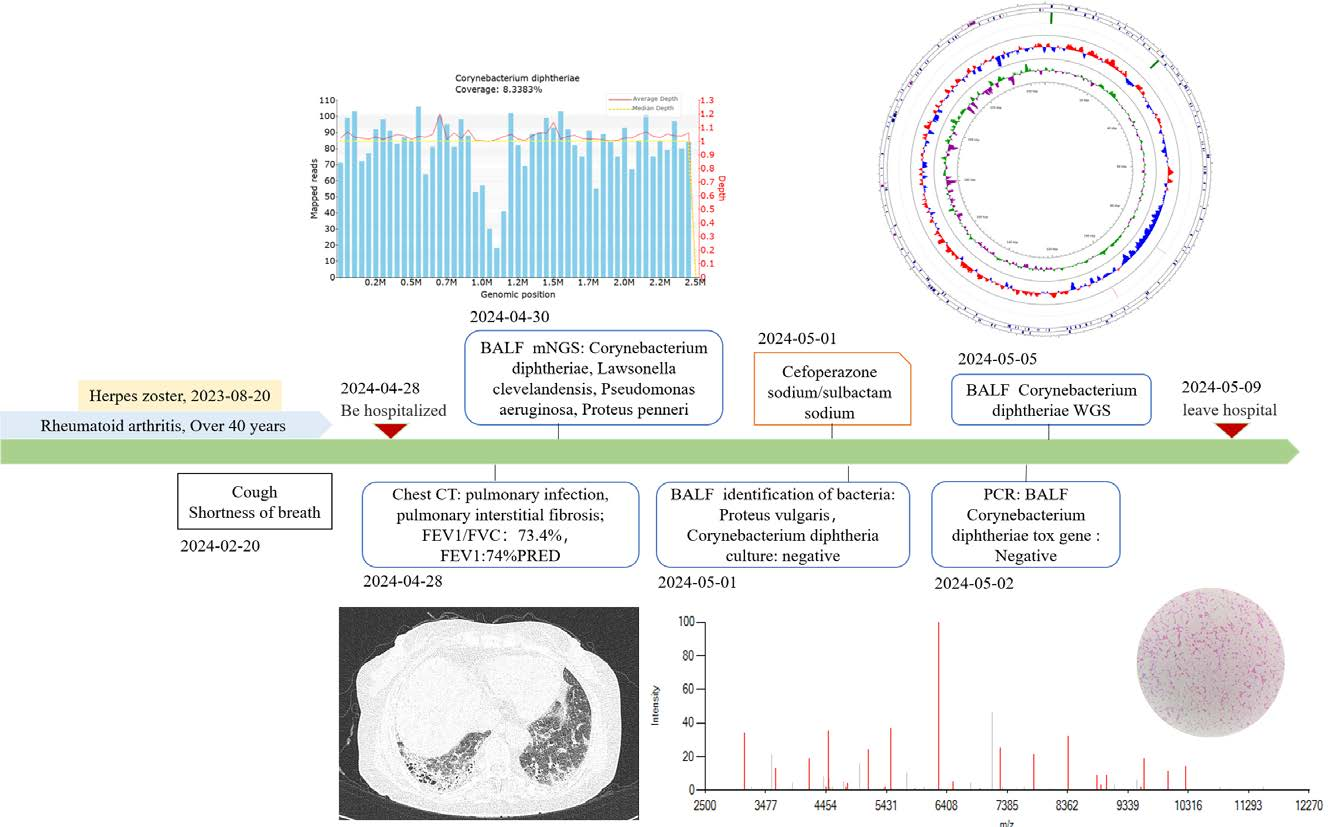
Course of disease.

A 71-year-old female patient with rheumatoid arthritis presented to the respiratory department of cough with shortness of breath for >2 months. Suffering from “herpes zoster” for half a year, without chills, fever, sputum cough, chest tightness, palpitation, chest pain, hemoptysis and other symptoms. Denied history of infectious diseases such as hepatitis, tuberculosis, typhoid, malaria; denied history of hypertension, coronary heart disease, diabetes mellitus; denied history of trauma; denied history of blood transfusion; denied history of poisoning; denied history of food and drug allergy; along with local vaccination including diphtheria-pertussis-tetanus vaccine. Spouse and children are physically healthy. The patient’s entire diagnosis and treatment process is illustrated in Figure 1.

### 2.2. Routine and radiographic examinations

On examination, Mean red cell hemoglobin concentration 310 g/L (normal value (316–354) g/L), hemoglobin concentration 106 g/L (normal value (115–150) g/L), red cell count 3.74 × 10^12^/L (normal value (3.80–5.10) × 10^12^/L), Thrombosis elastography test: blood clot mechanical strength 12.4 d/sc (normal value (4.60–10.90) d/sc), platelet function 71.30 mm (normal value (41.5–69.3) mm), Angle fibrinogen level 77.10 deg (normal value (46.2–72.5) deg), K fibrinogen level 0.80 min (normal value (1.2–3.6) min). Blood sedimentation: 41.0 mm/h (normal value (0–38.0) mm/h). Coagulation: Fibrinogen 4.11 g/L (normal value (2–4) g/L). Hepatic function: prealbumin 177 mg/L (normal value (180–390) mg/L), white ball ratio 1.12 (normal value 1.2–2.4), and albumin 37.5g/L (normal value 40–55). Transmission of 9 items: hepatitis B surface antibody positive. Blood lipid: low-density lipoprotein cholesterol: 3.36 mmol/L (normal value (1.5–3.3) mmol/L). Urine analysis: urinary protein positive. Pulmonary function test: FEV1/FVC:73.4%, FEV1: 74% PRED; mild impairment of restrictive pulmonary ventilation; moderate-severe limited small airway airflow, hyperventilation, ventilatory reserve function (77.6%), severely reduced diffusion function, FeNO: 8ppb. Diagnosis of chest computed tomography: nodular shadow of the left lung tip, pulmonary infection, and pulmonary interstitial fibrosis.

### 2.3. The examine of pathogeny

Auxiliary examination: automated identification was performed from colony growth of bronchoalveolar lavage fluid using matrix assisted laser desorption ionization-time of flight mass spectrometry (Bioyong Technologies Inc, Beijing, China): *Proteus vulgaris*. Bronchoalveolar lavage fluid Pathogenic Metagenomic Detection: *C diphtheriae* (sequence number 2168), *Lawsonella clevelandensis* (sequence number 1508), *Pseudomonas aeruginosa* (sequence number 1225), *Haemophilus influenzae* (sequence number 937), *Morganella morganii* (sequence number 693), *Proteus penneri* (sequence number 425), *P vulgaris* (sequence number 228), Human betaherpesvirus 5 (sequence number 9).

To confirm the presence of *C diphtheriae*, quantitative real-time PCR (qPCR) was performed to detect both genomic DNA and virulence genes. Results demonstrated genomic DNA positivity for *C diphtheriae* but negativity for virulence determinants. However, this study has limitations: insufficient sample quantity precluded repeated subculture attempts for bacterial isolation, and reliance on molecular methods alone currently precludes definitive determination of *C diphtheriae* viability.

### 2.4. Whole-genome sequencing

**Figure 2.**
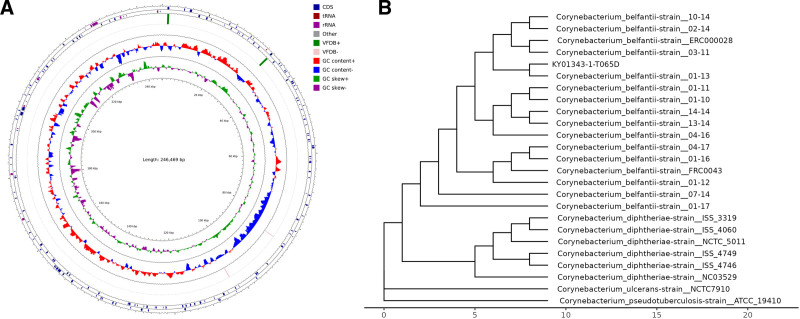
Chromosome and Phylogenetic relationships of *Corynebacterium diphtheriae* biovar Belfanti isolated. (A) Chromosome of *Corynebacterium diphtheriae* biovar Belfanti isolated. Circle diagram from inside to outside: 1. Genomic coordinates; 2. GC bias: used to measure the difference in the relative content of bases G and C in single-stranded DNA, GC skew + means that the content of G is greater than C, GC skew-means that the content of G is less than C; 3. GC content: GC content-indicates that the region is lower than the genome average GC content, and GC content + indicates higher than the genome average GC content; 4. Virulence factors: the distribution of virulence factors retrieved in the comparison of the genome and VFDB databases, and VFDB +/− indicates the positive and negative direction of genes; 5. Gene distribution: CDS and RNA distribution, orientation indicates gene direction; 6. The genes annotated in the circle map are drug resistance genes. (B) Phylogenetic relationships derived from the analysis of genomic sequences.

In order to truly reflect the genome information of *C diphtheriae* in clinical samples, the whole genome sequencing of pathogenic microorganisms was conducted directly on clinical samples. The complete, circular chromosome assembly of isolate T065D was 246,469 bp long. After assembling the data, the genome of the strain was obtained (NGDC GSA number: CRA020095). The genome circle map is shown in Figure 2A. The GC was 53.98%. Although the assembling genome length failed to cover the entire *C diphtheriae*, the results provide a useful reference for strain identification, analysis of drug resistance genes and virulence genes.

Tandem repeats finder searches for tandem repeats in DNA sequences. The prediction results are shown in Table 1. 3695 Long interspersed nuclear elements with transposition activity were detected. 188 tandem repeats, 114 MicroSatellites with 1 to 6 bases; 55 MiniSatellites with 10 to 60 bases were detected, suggesting that the strain may have a strong variation ability. The CRISPR prediction of the genome was performed by the CRISPRCasFinder software, A CRISPR sequence of 218bp in length was predicted (ContigID: k141_508), suggesting that the strain has immunity to the virus.

**Table 1:** Results of the virulence gene annotation.

Gene ID	VFDB ID	Gene name	Description	Pident (%)	Alignment length
KY01343-1-T065D_00073	VFG013693(gi:38233057)	sapD	Putative surface-anchored membrane protein [Surface-anchored pilus proteins (CVF510)] [*Corynebacterium diphtheriae* NCTC 13129]	98.1	160
KY01343-1-T065D_00070	VFG005353(gi:76787761)	plr/gapA	glyceraldehyde-3-phosphate dehydrogenase [Streptococcal plasmin receptor/GAPDH (CVF123)] [*Streptococcus agalactiae* A909]	63.8	58
KY01343-1-T065D_00041	VFG041304(gb YP_095978)	lirB	Dot/Icm type IV secretion system effector LirB [Dot/Icm (SS047)] [*Legionella pneumophila* subsp. pneumophila str. Philadelphia 1]	52.3	172

In the molecular function classification, the largest number of enriched genes are catalytic activity and binding. In the cellular component classification, the largest number of enriched genes are cellular anatomical entity and protein-containing complex. In the molecular function classification, the largest number of enriched genes are metabolic process and cellular process (Fig. S1, Supplemental Digital Content, https://links.lww.com/MD/O900). The PHI alignment resulted in 7 sequences of genes that play a role in the pathogen-host interaction, including 5 related sequences with reduced virulence, indicating a weak virulence between the isolate and the host.

A phylogenetic tree based on sequencing and database data was used to describe the evolutionary relationships among species with Neighbor-Joining. The results show that this strain isolated sequence is close to *C diphtheriae* biovar Belfanti strain and has evolutionary different from the Mitis, Gravis, and Intermedius (Fig. 2B).

**Figure 3.**
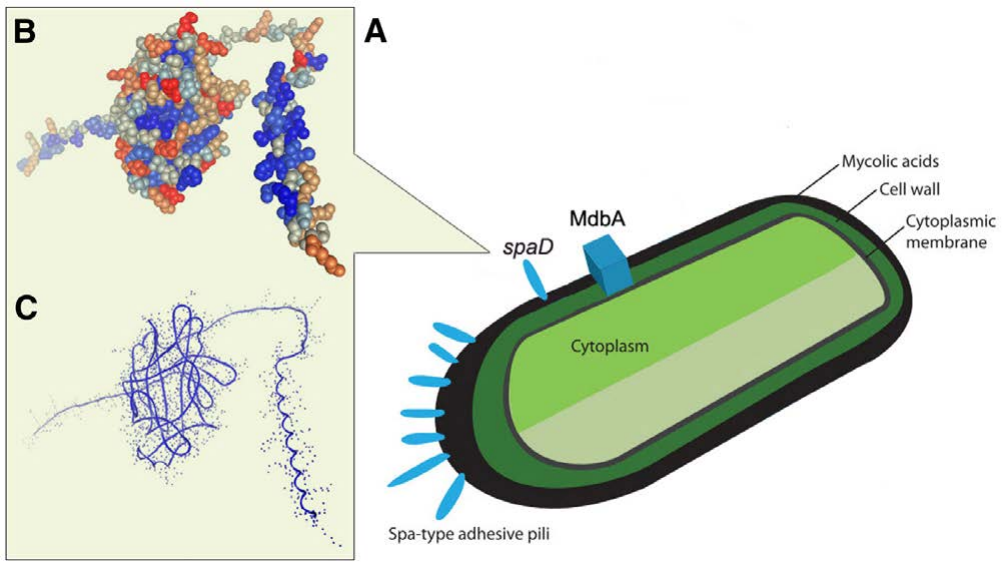
Niche and virulence factors of isolated strain. (A) Niche and virulence factors of isolated strain; (B) protein structure 3D modeling using the amino acid hydrophobicity annotation (The hydrophobic group is shown in blue and the hydrophilic groups in red); (C) protein structure 3D modeling using the amino acid secondary structure annotation; MdbA, thiol-disulfide oxidoreductase; (A) refer to the Andreas Tauch review.^[8]^

Using diamond software, the amino acid sequence of the target species was compared with the VFDB database, and the genes of the target species and their corresponding virulence factors were combined to obtain the annotation results. The results of the virulence factors annotated for this sample are shown in Table 1. 3 virulence factors are annotated, including sapD, plr/gapA, and lirB. Among them, the spaD virulence factor structure may be related to the specific adherence of corynebacteria to human pharyngeal epithelial cells, but the specific function of SpaD remains unknown^[7]^ (Fig. 3A).

3D modeling of the spaD amino acid sequence using swiss-model software revealed the protein structure with a distinct transmembrane segment, a carbon terminal transmembrane segment and a hydrophobic segment^[9]^ (Fig. 3B). The middle amino acid section forms a cylindrical structure with hydrophilic group wrapping, and the nitrogen end also has a partial hydrophobic group, which may be related to the binding function of membrane or other proteins (Fig. 3C).

At the onset of admission, the patient received intravenous infusion of cefoperazone sodium and sulbactam sodium at an initial dose of 4 g twice a day. Concurrently, doxofylline was prescribed at an initial dose of 0.2 g/day. The patient continued to receive aerosol inhalation of Budesonide Suspension for Inhalation 2.0 mg/day and levosalbutamol hydrochloride 0.63 mg/day. On May 9, 2024, the patient had improved chest tightness and shortness of breath, no other special discomfort, and stable vital signs after physical examination.

## 3. Discussion and conclusions

In this case, a patient with rheumatoid arthritis had cough without obvious inducement, and computed tomography indicated pulmonary interstitial fibrosis. In order to identify whether fibrosis was caused by autoimmune system disease or other infectious factors, Pathogenic Metagenomic Detection was performed for the patient. Among them, the non-toxigenic *C diphtheriae* biovar Belfanti strain was isolated. In the culture identification experiment, the mass spectrum of this bacterium was *P vulgaris*, which was different from that of Pathogenic Metagenomic Detection. The reason may be that ① the biovar Belfanti is not capable of nitrate reduction, unlike the other strains of *C. diphtheriae*^[1]^; ② *P vulgaris* has a growth advantage over *C diphtheriae*. During the verification of the PCR method, it was also found that this bacterium did not have common diphtheriae virulence genes, so there is the possibility of missing detection in the culture identification and PCR link. Because the bacterium is sensitive to penicillin, macrolides and other drugs, so it is difficult to be found and isolated. *C diphtheriae* was effectively inhibited after antibiotic treatment.^[10]^ In this case, pulmonary interstitial fibrosis was thought to be associated with autoimmune disease and was not associated with *C diphtheriae* infection. However, the detection of this case indicates the spread of the *C diphtheriae* biovar Belfanti strain in the Chinese population. Although there are no clear virulence genes and drug resistance genes, and the pathogenicity is not strong, the strain has high transposing activity and strong infectivity. However, the possibility of development into pathogenic strains cannot be ruled out and is worthy of attention.

Historically, the exotoxin produced by *C diphtheriae* has been recognized as the primary pathogenic factor in diphtheria, with toxigenic strains typically manifesting as gray-white pseudomembranes in the pharynx, larynx, and nasal cavity. However, recent studies increasingly report infections caused by non-toxigenic strains, which frequently present as bacteremia or endocarditis with high case-fatality rates. Concurrently, the isolation rate of non-toxigenic strains has shown an upward trend. Notably, in countries with high diphtheria vaccination coverage, infections attributable to non-toxigenic strains have become significantly more prevalent. In this case, the patient exhibited neither pseudomembranous lesions nor bacteremia, demonstrating a subclinical presentation with high potential for diagnostic oversight. Fortunately, the isolated strain exhibited attenuated virulence and limited invasive potential, resulting in no significant clinical harm to the patient.

During etiological diagnosis, given that *C diphtheriae* biovar Belfanti is nitrate reduction-negative and lacks virulence genes, it is recommended to integrate culture-based identification with molecular methods (e.g., PCR or sequencing) for comprehensive evaluation. Molecular diagnostic results should be prioritized to some extent, particularly for detecting virulence determinants. Molecular diagnostic results should be prioritized to some extent in clinical decision-making.

## Author contributions

**Conceptualization:** Huajie Xie, Wanlin Na, Youde Liu, Qin Jia, Wei Sun, Yanyan Wang, Yuan Liu.

**Data curation:** Huajie Xie, Youde Liu, Qin Jia, Wei Sun, Yanyan Wang.

**Formal analysis:** Wanlin Na.

**Funding acquisition:** Kai Chang.

**Investigation:** Kai Chang, Huajie Xie, Ming Liu.

**Methodology:** Ming Liu.

**Writing – original draft:** Kai Chang.

**Writing – review & editing:** Kai Chang, Yanyan Wang, Yuan Liu.

## Supplementary Material
