# Supplementary material for: A non-toxigenic Corynebacterium diphtheriae biovar Belfanti isolated from a patient with rheumatoid arthritis in China: Insights from a comprehensive genome-based analysis
Source: Medicine (Baltimore). 2025 May 16;104(20):e42467. doi: 10.1097/MD.0000000000042467 (PMC12091665; doi:10.1097/MD.0000000000042467)

Supplement figure 1. Results of Cluster Analysis on the Data

A. GO function classification; B. KEGG function classification; C. COG function classification; D. PHI annotation-classification statistics of phenotypes in genes that affect pathogen host interactions.


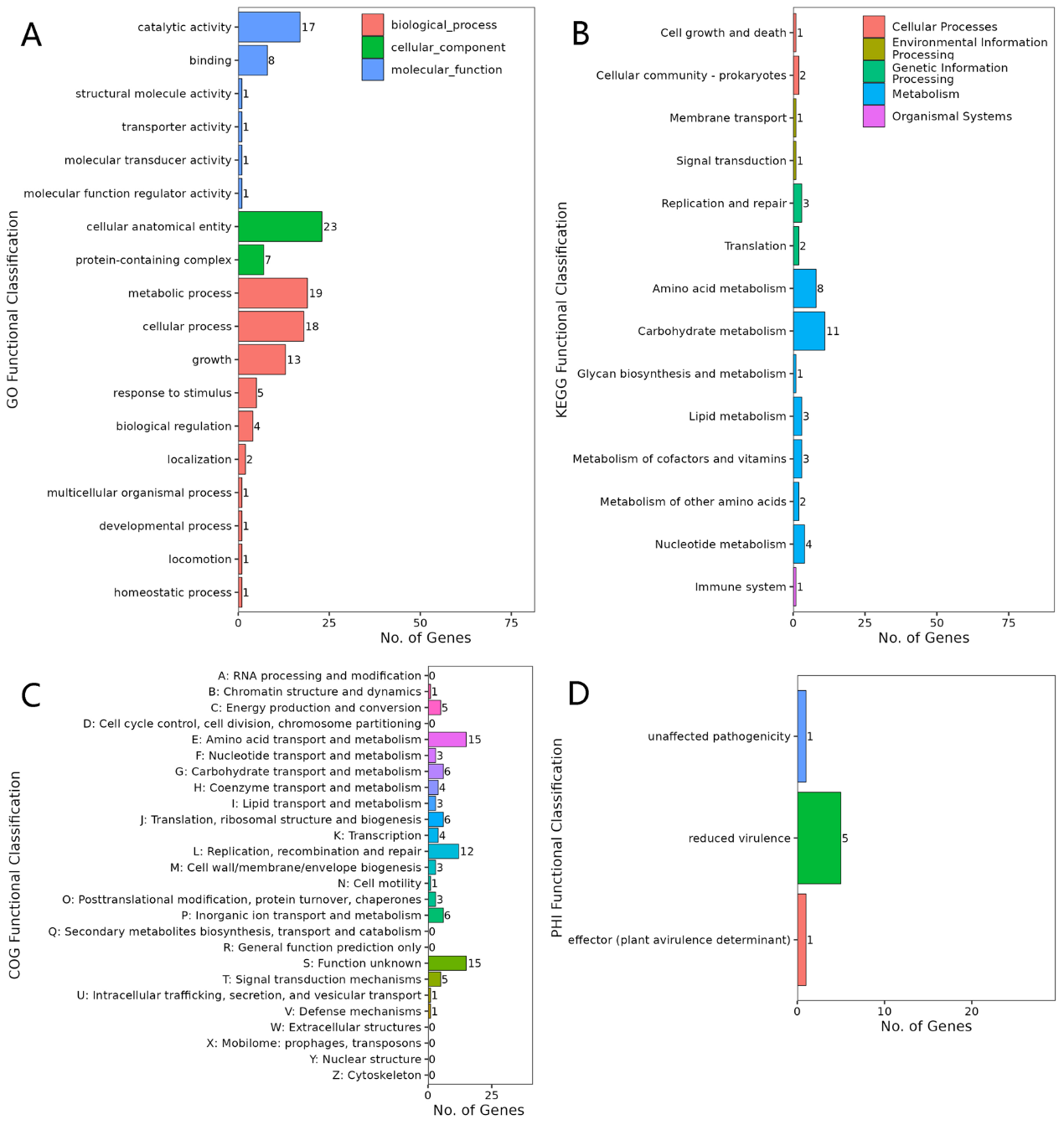

Supplement: Supplementary file 1 [file medi-104-e42467-s001.docx]
